# Aluminum Base

**Published:** 1873-02

**Authors:** C. Sauer

**Affiliations:** Berlin.


### ARTICLE V.

### *Aluminum Base.*

BY C. SAUER, BERLIN.

Read before the Association of German Dentists, held in Vienna, 1872.

At the last meeting I gave you some account of my experiments with aluminum, and especially the examination I made in regard to its durability in the mouth. At this meeting I intend to explain my mode of working this metal. During the past year I have been enabled to make considerable improvement, and also to simplify it.

The process I will give you proves that whatever can be made with rubber, (caoutchouc,) can also be made with aluminum. Experience can alone prove whether aluminum can be considered equal to the rubber. I hope that my communication will have the effect of bringing this metal more into use, and thus we will be enabled in a variety of ways to examine aluminum and its alloys.

The process of working aluminum very much resembles that of rubber. As I formerly informed you, I make my casting upon the original model, and at the same time, by the casting, bind the metal to the teeth. The apparent simplicity of this work may lead subsequently into error. I will not weary you with a recital of the progressive steps taken in this investigation, but will only give you the advantages which I at present find in this process. There will be found many things incomplete in its practical working; but these, I hope, will be removed in time.

The principal labor in putting up an aluminum piece is the casting. If I intend to accomplish this perfectly I must strictly regard the melting point of the metal. The temperature of the material composing the cast model, as well as the teeth, must approach that of the melting point of the aluminum. The filling of the flask must shrink as little as possible in the glowing heat to which it is subjected. Great care is necessary not to allow the metal to decompose, to which it is very liable by the chemical changes produced by the heat.

Plaster, an excellent article for other purposes, is bad for this, as it falls to pieces in the heat, and would also cause chemical decomposition in the aluminum. But with substances containing gravel and pumice stone, it stands well at a high temperature. Clay has, by its composition, so near a relation to aluminum that it can, for this reason, be used as a filling for the flask. Its resistance to the effects of high temperature is an important consideration. For the same reason I consider graphite to be valuable. As the graphite contains carbon, it is liable to enter into combination with different gasses during the time subjected to the glowing heat, and they are injurious to the aluminum, oxygen being one of these.

The composition as I use it consists of—Plaster, one part; Pumice, one-half part; Clay, one-sixth part; Graphite, one-sixth part.

This composition seems to me to answer the best purpose. It contracts very little in the glowing heat and casting, and becomes in the heated composition very fine and tender.

I must here, at the commencement, speak in opposition to the opinion that the cast aluminum plates do not fit in the mouth. They are as well adapted as the rubber work, the atmospheric cavity operating as well in one as the other.

The temperature of the teeth is, of course, the same as that in the filling of the flask, which, in casting, is red hot. Between the hot teeth and the melted aluminum there is probably an important difference of temperature unfavorable to the teeth. The pressure of the steaming aluminum on the glowing teeth is also unfavorable. I am forced to these suppositions by the result of some experiments. The teeth exhibited fissures after the casting, during the finishing process, or were cracked in the mouth without apparent cause. When the reason for this fault was clear, I attempted to prevent it by arresting the flow of aluminum just before it came in contact with the teeth, and by this means cooling it. The size of the casting (gussraum) for the plate involved me in other doubts. The casting plates must be as thin as possible, and yet must be free of holes. These will result if the pattern and the casting plates are too narrow or too unequal. I must, therefore, test the thickness of the pattern; the casting can then be made as thin as possible, and free of holes. The composition of the filling of the flask is therefore of great consequence, as it in all cases must show the same contraction. I make my pattern in this way—that is, the diameter is from 1 to 1 1-8th m.m., the casting is about the thickness of 1 m.m., and can be reduced at pleasure by filing. I roll plates of lead to 3-4 m.m. It is very difficult to make them of greater thickness. The strength is increased by rolling tin to the thickness of 1 1-8th m.m. The number of sheets of tin foil to be placed must be in accordance with the depth of the jaw. I make use of few in a small vault, many in a large one. The plates of tin are first placed on the model, to be followed by the lead plate. This plate is to be placed on as neatly as possible, at first with the finger, and then with a piece of wood rounded at the end.

Before I proceed further with the process I must describe the flask, the apparatus to warm it, and the one to mould it.

The flask is of the same cylindrical shape with which you are familiar in rubber work. It consists of four pieces; two of them are cylinders of different heights, and open at both sides, with two covers. The difference between these and rubber flasks consists in a hole in the side wall of the higher cylinder for the mould, and a tube placed upon the hole for moulding. The covers of the flask have on two sides opposite each other joined pieces, somewhat resembling a tuning fork. These joined pieces must stand exactly opposite to each other when the flask is closed. These are for two screws to bring together the covers of the flask. The screws end on one side with a button, and on the other with a button. The latter runs in a box. The button and the box afford resistance when the different parts of the flask are brought together. The lower cylinder serves for the reception of the model, and has always on the under side a ledge. The higher cylinder gives position to the piece. The filling tube, which is placed to one side, is split its whole length, the upper and under parts being put together by a screw. The screw at the same time serves to secure the filling tube against the moulding hole of the flask. The filling tube has on the outside a small rim, and upon this is loosely placed a box. The channel is situated below the filling tube. The flask has on the outside of the moulding hole the thread of a screw. The tube by the rim is screwed by the box toward the flask, so that they form a continuation. A short funnel is screwed on the split filling tube at the upper part. This has on the inner side a width of 8 m.m., the thickness is 3 m.m., and has, excluding the funnel, a height of 8 m.m. The moulding hole of the flask has a conical shape toward the inner side. The button of the aluminum will, by this arrangement, be easily removed. This button has to be sawn off on the inner side of the moulding hole. I speak of this especially, because I always leave a moulding head (button) in the filling tube in proportion to its height. It gives the pressure under which I mould. I regulate the pressure in accordance with the height of the moulding head or button. I would especially call your attention to the width of the filling tube, as it enables us to use less material in the melting. All the experiments which I made, either with wide or narrow filling tubes, taught me that the wide would not produce such good results. The flask, with the filling tube and funnel, are of cast iron, and the two screws and box of malleable iron.

The heating of the flask is done in an oven used for heating irons large enough to accommodate two flasks at the same time. In it is a box of cast iron, open on the upper and under parts, with feet to support it. On the inner side of the box are two rims opposite to each other, on which to place the flask. The flask is laid down with the two covers. These are formed by the cover of the flask. This oven is covered on the outside by chamotte, (a very hard stone used for stoves,) the object being to confine the heat as much as possible. The oven is closed on the upper part by a plate of iron and chamotte. The whole is to be heated with gas. The cover has an opening for the moulding funnel, which serves at the same time the purpose of a chimney. The funnel is placed above this opening to facilitate the moulding. The covers and all the pieces of the flask have handles. This gas moulding oven or furnace I can also use for melting gold, &c., and I at one time melted in it seven ounces of brass. The melting furnace consists of a pipe of malleable iron, 26 ctm., and a width of 12.3 ctm. It is placed upon three feet of hoop iron 13.3 ctm. in length. The oven has on the upper and under part a rim of the diameter of 8 ctm. On the inner side is placed a compound of chamotte, mud and pieces of brick. This forms nearly a cylinder, and is about 13.5 ctm. At this place on the inside are three iron wires to form a triangle. If aluminum is to be melted, a refining pot is placed upon the upper rim of the oven with one mark of the metal. Upon this upper rim is placed a chimney of conical shape, made of sheet iron, 31 ctm. in height. Its diameter at the upper part is 4 ctm. This chimney is covered by chamotte three-fourths of its height. The funnel used is put on the oven to a height of 7 ctm. If you are moulding you have to cover the oven with the chimney.

I work in, aluminum in the following manner: I make two models of one impression. One of these is of plaster, clay, pumice stone and graphite; the second only of plaster. The teeth are ground, as indicated by the plaster model, put together on the base plate. They are secured by wax, and the bite taken. The piece is then placed with wax upon the plaster model. It is then placed in the flask with the previously described composition of plaster, clay, pumice and graphite. The back part of the model must be opposite to the moulding hole. The upper part of the flask is now put on the under part, and the wax patterns for the base are placed in, extending in a forked direction from the casting aperture to both sides of the arch. They are round on the moulding hole, and become flat on the end, but are here broader than on the former. They are then fastened on both sides and flasked, and the cover placed on. This mode is adopted where the teeth are fitted directly on the jaw, but if not, then the manipulator must proceed as in rubber, placing the model in the under part of the flask, and the work with the teeth in the upper. If teeth with gums are used, they must close on the jaw, or they would readily spring. The more securely the teeth are placed in the plaster composition, the less likely they are to spring. The surest way is to place them on the model with plaster. Air openings should be made from the base plate to the edge of the flask. Use iron wire to make air openings from the interspace to the under part of the flask. The wire is to be removed and the openings filled with wax. All the other openings are cut after the flask is separated. The base plate and wax are now to be removed from the model. To avoid danger of springing, a wire of aluminum is placed at the distance of a half to one m.m. from the pins. This is secured in the composition of plaster. If the bite is not very high, I do not place in this wire, but if the bite is very high, as in gum teeth, several wires are used, one on the other. A wire may also be run in the vault below the gum teeth, in order, in the heat, to equalize the different thicknesses of enamel. The wax patterns are finally removed by boiling water, the part screwed on, and both parts of the flask slightly warmed. To accomplish this they are very gradually heated, the temperature being slowly raised for three-quarters of an hour. At last there must not be steam enough escape to moisten the surface of a mirror. When they are thus well prepared they are screwed together and placed in the warm oven and heated over the burner. The flask must still retain its black color. The furnace is now heated, and the aluminum, with pulverized coal, is placed in the graphite refining pot, covered by the chimney. This flue, pulverized coal, I have made use of since it was introduced by Franz Schulze, of Berlin, a manufacturer of alum inum bronze. When the aluminum begins to melt, the chimney is removed and the metal gently stirred until it is thoroughly melted. Then the slacks are removed, and the melted mass poured out. The flask should be about blood heat. My experience teaches me that this temperature is the best to get a good mould, and without endangering the teeth. The melted metal comes in contact with the wire in its passage to the teeth, by which it is prevented from too rapidly encroaching upon them, and is also cooled to some extent. If the melted metal is permitted to go direct to the pins of the teeth, these will, by the superior power of conducting heat, produce small fissures in the teeth before the metal has reached them. The flask must remain in the warm oven after the moulding to become thoroughly cooled. It is then to be separated, and the work cut out with a saw.

The finishing of this work is done in the same way as the rubber. If I polish, which I seldom do, I use steel, with equal parts, by weight, of rum and oil.

Mending an aluminum case is only possible by soldering. The difficulty of accomplishing this is great, as all the aluminum solders decompose very rapidly in the mouth, and form an alloy with the pins more rapidly if they contain zinc or tin. The teeth will drop very soon from the soldered part. Both of these metals are to be avoided, all the more as the aluminum readily unites with an alloy. To avoid decomposition, I considered it best to take such metals which have in their electrical affinity the greatest contrast to aluminum. After many experiments with different kinds, I used for a long time a solder of gold, brass and aluminum. I thought I was obliged to have recourse to the zinc in the brass, but I was always disturbed by the decomposing quality. I observed that platina and aluminum alloy very easily. I, therefore, now use as a solder a composition:

Gold, 2.9 parts; Platinum, 0.1 part; Copper, 2.0 parts; Aluminum, 10.0 parts.

This solder probably remains good in the mouth on account of the percentage of platinum, and does not, therefore, readily form an alloy with the pins. With it I have soldered platina silver wire on aluminum.

In forming the composition, the gold, copper and platina must first be thoroughly melted. I do this with coal powder in a small refining pot in the melting furnace, placed on the triangle and covered with the chimney. When it is melted I take off the chimney and place the weighed aluminum in a graphite refining pot, with the powdered coal in the upper part of the melting furnace, and again cover with the chimney. When the aluminum is melted I pour it in the refining pot below, filled with the composition. This is to be stirred. From the refining pot I pour this solder upon an iron, or what is better, a porcelain plate, and make it flat. If I want to solder I take from it a small piece.

I need for soldering a burner, tongs to hold the work, a thin piece of iron, easily bent, to place under the work to keep it in place during soldering, a composition of plaster and pumice-stone to flask it, and soldering iron. This consists of a pointed piece, bent in the form of a screw. If it is desired to solder teeth, I protect them with a plate of aluminum and bend the pins against it. This plate, pins, and the part to be soldered are to be cleaned from all foreign metal. The teeth are then placed on the piece with wax, and flasked with plaster and pumice-stone composition. In the plaster is placed a strip of plate iron, resting against the teeth. Plaster must not be placed beneath the teeth to be soldered. Finally, a piece of iron plate is bent to the shape of the jaw and secured below it. After securing the piece to the teeth to be soldered, the wax is to be removed. The whole is to be held above the flame of the burner until it is glowing. Solder is then placed on the point to be soldered. Where it is soft, carefully rub with the soldering iron against the gum and protecting plate. In soldering it is well to bear in mind two things: 1st, That the teeth do not become too glowing. 2d, That the first piece of solder is not too large and placed deep in the part to be soldered. Finally, I solder the protecting plate and the work on the back of the gum.

Recently I have used, instead of aluminum, a composition of aluminum and silver and one of aluminum, silver and platina. I found that when the casting has been made too hot, decomposition has taken place on the surface, after having been worn for a time. This, however, did not spoil the work. I therefore tried a composition of 95 aluminum and 5 of silver in hopes of better results, the silver being more elastic than aluminum. As silver alloys readily with the platina pins, I placed in a small quantity of platina to prevent it. The results were very good. In a composition in which platina is combined, I could fasten clamps of silver and platina on the cast. This solder can also be used for mending.—*Dental Times.*

